# Timing for closed reduction procedure for developmental dysplasia of the hip and its failure analysis

**DOI:** 10.1186/s12891-020-03635-1

**Published:** 2020-09-14

**Authors:** Zhiqiang Zhang, Hao Li, Hai Li, Ziming Zhang

**Affiliations:** 1 Department of Orthopedics, National Children’s Medical Center & Children’s Hospital of Fudan University, 399 Wanyuan Road, Shanghai, 201102 China; 2 Department of Orthopedics, Shanghai Sixth People’s Hospital, School of Medicine, Shanghai Jiaotong University, 600 Yishan Road, Shanghai, 200233 China; 3 Department of Pediatric Orthopedics, Xinhua Hospital, School of Medicine, Shanghai Jiaotong University, 1665 Kongjiang Road, Shanghai, 200092 China

**Keywords:** Developmental dysplasia of the hip, Closed reduction, Avascular necrosis, re-dislocation, Residual acetabular dysplasia

## Abstract

**Background:**

It remains controversial whether the older age to perform closed reduction (CR) procedure for developmental dysplasia of the hip (DDH), the higher incidence of complications. The aim of this study is to evaluate the midterm outcome of CR for DDH among different age groups, and to analyze and identify risk factors for the failure of this procedure.

**Methods:**

Clinical data of 107 DDH patients, who received CR, were retrospectively reviewed. Data were divided into three groups according to initial treatment age (Group I: younger than 12 months; Group II: 12 months to less or equal to18 months; Group III: older than 18 months). The presence of avascular necrosis (AVN), residual acetabular dysplasia (RAD), re-dislocation, and further surgeries (FS) were observed. The risk factors were identified for those outcomes aforementioned using univariable logistic regression models. For identified risk factor age, pre-op acetabular index (AI) and post-op AI, their prediction of CR failure were evaluated by receiver operating characteristics curve (ROC).

**Results:**

A total of 107 patients (156 hips) undergoing CR procedure were evaluated with a median age at initial reduction of 13.0 ± 5.4 months (range, 4 to 28 mo). Mean follow-up time in this study was 6.7 ± 0.8 years (range, 3–8 years). The incidence of AVN, RAD and re-dislocation was 15.4% (24/156), 17.3% (27/156) and 14.7% (23/156) respectively. For AVN, RAD and re-dislocation, the significant risk factors are pre-op IHDI IV (*p* = 0.033), age ≥ 18 months (*p* = 0.012), and pre-op IHDI IV (*p* = 0.004) and walking (*p* = 0.011), respectively. The areas under the ROC curve of each type of failures were 0.841 (post-op AI), 0.688 (pre-op AI) and 0.650 (age).

**Conclusions:**

Severe DDH patients older than 18 months with CR procedure may result in a high risk of RAD complication. Re-dislocation is significantly associated with pre-op IHDI IV and walking. Patients, who are older than 12.5 months or have a pre-op AI of 38.7° or a post-op AI of 26.4°, are also more likely to fail of CR procedure.

## Background

Developmental dysplasia of the hip (DDH) is the most common developmental malformation affecting children’s hips. The widely adopted principle for management of DDH is that a concentric reduction should be obtained and maintained through the intervention period as early as possible [1, 2]. Closed reduction (CR) of the hip is performed on patients who failed to achieve stable reduction with Pavlik harness, or as the primary treatment option for patients with late diagnosis [3, 4]. Although this procedure generally achieves satisfactory outcomes, CR procedure may also lead to a number of adverse complications, including iatrogenic avascular necrosis (AVN), re-dislocation and residual acetabular dysplasia (RAD), which might need further surgeries (FS) to address the problem. Previous studies reported that increased age at the time of CR predicted a higher rate of complications or further corrective surgeries [5–7], while others not [8, 9]. Moreover, it still remains controversial whether CR or open reduction (OR) should be adopted for children approaching or older than 18 months at the time of first diagnosis, especially for the severe dislocated cases. Balancing the advantages and disadvantages of different treatment options based on evaluating the risks of complications for each, will help to achieve a better patient outcome. The aim of this present study was to evaluate the effect of CR among different age groups, to identify the risk factors of complications of CR and to identify the ones significantly associated with CR failures.

## Materials and methods

With the institution Ethics Committee approval (XHEC-D-2020-014), a retrospective review was performed for 107 pediatric patients (95 female and 12 male) with the diagnosis of DDH from January 2011 to December 2013, who underwent CR and cast fixation and met our inclusion criteria. The inclusion criteria were: 1) At least 36 months follow-up time and complete medical records. 2) Diagnosed with unilateral and/or bilateral DDH with International Hip Dysplasia Institute (IHDI), III, IV grade, or Tönnis III, IV grade, without any treatment before. 3) Successful CR at initial attempt. Patients were excluded if their follow-up time less than 36 months, pathological or other secondary hip dislocation, dysplasia of the hip without hip dislocation or unsuccessful CR initially (post-operative MRI indicating dislocation of the hip for 11 patients).

**Fig. 1:**
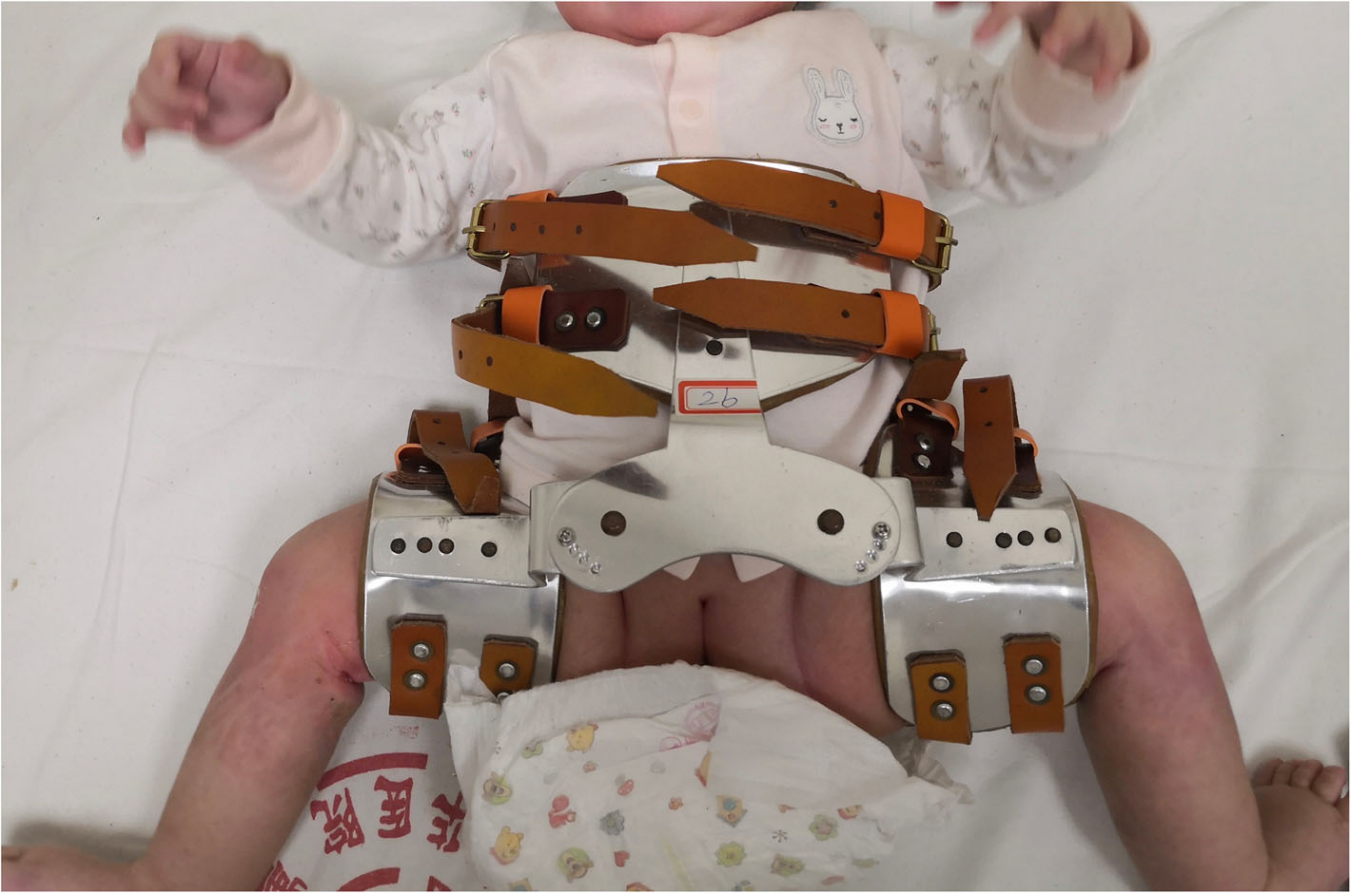
Orthosis used after cast removal. The orthosis contained four holes with cap nuts and was adjusted timely according to our protocol of 1-2-2-2-month

CR under fluoroscopic guidance was performed under general anesthesia in all cases. After the percutaneous adductor tenotomy, closed reduction was performed according to a routine procedure. Namely, the hip was reduced by placing it in flexion nearly 100 degrees and gradually abducting it to the position of stability (nearly 45 to 65 degree). Then, a hip spica cast was fixed in a human position with a gentle posterior mold. MRI examination was carried out under sedation within 24 h postoperatively. The spica cast was maintained for 3 months. Plain radiography of the pelvis was taken monthly. After three-month immobilization, the cast was removed and an adjustable abduction orthosis was applied for 7 months. The orthosis contained four holes with cap nuts and was adjusted timely according to our protocol of 1–2–2-2-month (Fig. 1).

All patients were divided into 3 different groups based on the age at initial time of CR treatment. Group I (the initial treatment age younger than 12 months), Group II (age 12 months or older but younger than 18 months) and Group III (age older than 18 months).

The medical data of all patients were collected. Anteroposterior x-ray films were obtained pre- and post-operatively till the final follow-up. Radiological data were evaluated which included the ossification centers of femoral heads, AI, Tönnis and IHDI grade; Complications included re-dislocation, RAD and AVN were detected. Re-dislocation is defined as hip dislocated on MRI during casting time or on X-ray when finished the CR. RAD is evaluated by AI (AI> 28° 1 year following CR or > 25° two to 4 years after CR [10]).The presence or absence of AVN based on the final follow-up was determined by Salter et al. [11], with a simple “yes” versus “no” to reduce subtype variability. FS of open reduction (OR) and osteotomy were warranted when RAD or re-dislocation existed. Failure was defined as either an OR at any time and/or AVN at the final follow-up. All measurements and evaluations were made independently by two authors who were not involved in the clinical care and without the prior knowledge of the outcome of the treatments.

Continuous variable was analyzed by Kolmogorov-Smirnov test to assess for normality. Comparisons of three groups in terms of AI, time of splint immobilization and follow-up time were performed by ANOVA. The chi-squared test was used to compare categorical variables (i.e. walking, ossific nucleus, Tönnis and IHDI grade, AVN rate, RAD rate, re-dislocation rate and FS rate). Furthermore, univariable logistic regression was performed to evaluate the relationships among the prereduction factors, including age groups, walking, ossific nucleus, preoperative AI, Tönnis and IHDI grades. The ROC curve was used to evaluate the sensitivity, specificity and diagnostic accuracy of the identified risk factors (age, pre-op AI and post-op AI) in predicting CR failure. A *p* value < 0.05 was considered significant for all statistical tests. Statistical analysis was performed using SPSS 19.0 (IBM, America).

## Results

**Fig. 2:**
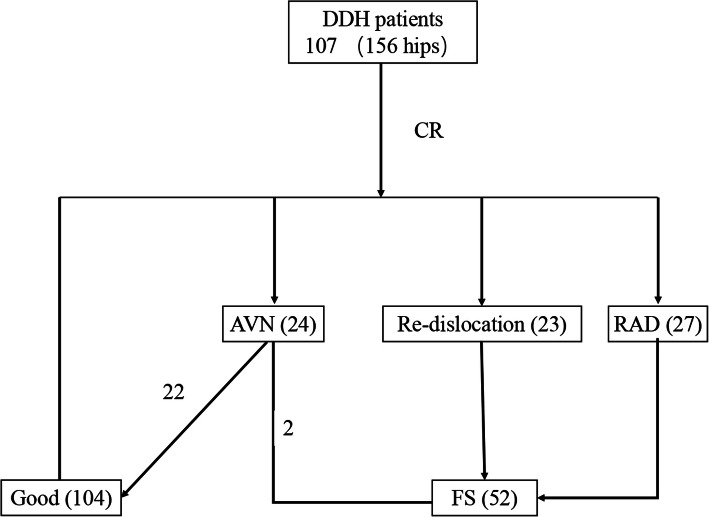
Flowchart of clinical outcomes of DDH patients treated by CR. 107 children (156 hips) with DDH received CR. There were 104 hips achieved good outcome, FS were performed on 52 hips (33.3%), of which 23 (14.7%) due to hip re-dislocation, 27 (17.3%) of RAD, and 2 (1.3%) of AVN

Total 107 children with DDH had received CR followed by plaster and orthosis fixation. Among them, 58 were unilateral DDH patients and 49 were bilateral who was at least present with one side hip dislocation. Mean age at initial treatment was 13.0 ± 5.4 months, ranging from 4 to 28 months. Mean follow-up time in this study was 6.7 ± 0.8 years (range, 3–8 years). A visible ossific nucleus of the femoral head was present in 103 hips (66.0%). Mean pre- and postoperative AI were 37.9° (range, 26.2°-49.7°) and 27.1° (range, 14.6°-37.6°), respectively. Subsequently, FS were performed on 52 hips (33.3%), of which 23 (14.7%) due to hip re-dislocation, 27 (17.3%) of RAD, and 2 (1.3%) of AVN. The incidence of AVN of femoral head was 15.4% (24/156) (Fig. 2).

### CR of DDH at different age groups

The influence of age at the beginning of treatment for DDH with CR is presented in Table 1. There was no significant difference in the general clinical attributes (sex, side, femoral head, splint time and Tönnis grade) among the three groups. Compared with Group I, the number of cases of pre-op IHDI grade was significantly different in the other two Groups, while the number of cases of pre-op Tönnis grade was not. However, postoperatively, no significant difference was found between IHDI and Tönnis grade. As for the number of cases for pre-op AI, there was no statistically difference among the three groups, while the case for post-op AI was significantly higher in Group II and III compared with Group I. Among the post-op complications, only the number of RAD cases in Group III was significantly higher than the other two groups. Moreover, significant difference was found between Group III and other two groups in FS rate.

**Table 1:** Demographic data with reference to age when DDH management of CR was initiated

	Group I	Group II	Group III
No. of hips (cases)	71 (45)	50 (37)	35 (25)
Age at present^0^(months)	7 (1.8)	15 (1.6)	19 (2.1)
range	4–10	12–17	18–28
Sex (girls: boys)	39:6	33:4	23:2
Side (unilateral: bilateral)	19:26	24:13	15:10
Walking	0	33 ^**b**^	35 ^**b**^
Femoral head (yes: no)	20:51	48:2	35:0
Orthosis time^0^ (mons)	6.6 (2.1)	5.9 (2.1)	6.8 (1.9)
Pre-op Tonnis			
I	8	1	2
II	3	7	2
III	34	13	9
IV	26	29	22
Pre-op IHDI			
I	2	0	1
II	11	10	2
III	48	19	16
IV	10	21	16
Pre-op AI^0^	36.9 (6.9)	38.1 (4.5)	39.2 (5.8)
Post-op AI^0^	25.5 (5.0)	27.9 (5.1) ^**b**^	29.6 (5.5) ^**b**^
Post-op Tonnis			
I	47	35	25
II	11	5	6
III	7	10	1
IV	6	0	3
Post-op IHDI			
I	45	34	22
II	11	8	9
III	11	8	4
IV	4	0	0
AVN (%)	10 (14.1%)	9 (18.0%)	5 (14.3%)
Re-dislocation (%)	9 (12.7%)	8 (16.0%)	6 (17.1%)
RAD (%)	6 (8.5%)	7 (14.0%)	14 (40%) ^**ab**^
FS (%)	15 (21.1%)	15 (30.0%)	22 (62.9%) ^**ab**^

Group I (< 12 months), Group II (12 months to ≤18 months), Group III (> 18 months)

*AI* Acetabular Index, *RAD* Residual Acetabular Dysplasia, *FS* Further Surgeries, *IHDI* International Hip Dysplasia Institute, *AVN* avascular necrosis.

^0^ values of mean (SD)

^a^: *p* < 0.05 compared with Group II

^b^: *p* < 0.05 compared with Group I

### Univariable logistic regression of prognostic factors for different outcomes

Univariable logistic regression was used to develop models predicting the potential Odds Ratio (OR) for each risk factor (Table 2). For AVN, pre-op IHDI IV was found to be a significant risk factor (OR: 2.524; CI:1.076–5.919; *p* = 0.033). Furthermore, by stratified analysis, early CR did not reduce the risk of AVN. For RAD, age ≥ 18 months was found to be a significant risk factor (OR: 4.000; CI:1.361–11.755; *p* = 0.012). For re-dislocation, pre-op IHDI IV and walking were found to be significant risk factors (OR: 4.211; CI:1.585–11.245; *p* = 0.004 and OR: 3.551; CI:1.339–9.412; *p* = 0.011, respectively). The significant risk factor of FS was pre-op IHDI III (OR: 27.506; CI: 3.234–233.919; *p* = 0.002).

**Table 2:** Odds Ratio Estimates for Risk Factors of Several Outcomes

Outcomes	Risk Factors	Odds Ratio	(95% CI^a^)	P
AVN	Pre-op IHDI IV	2.524	1.076–5.919	0.033
Re-dislocation	Walking	3.551	1.339–9.412	0.011
	Pre-op IHDI IV	4.211	1.585–11.245	0.004
RAD	age ≥ 18 months	4.000	1.361–11.755	0.012
FS	Pre-op IHDI III	27.506	3.234–233.919	0.002

*AI* Acetabular Index, *RAD* Residual Acetabular Dysplasia, *AVN* Avascular Necrosis, *IHDI* International Hip Dysplasia Institute, *FS* Further Surgeries, ^a^*CI* confidence interval.

### ROC to evaluate the prediction of CR failure for each identified risk factor

**Fig. 3:**
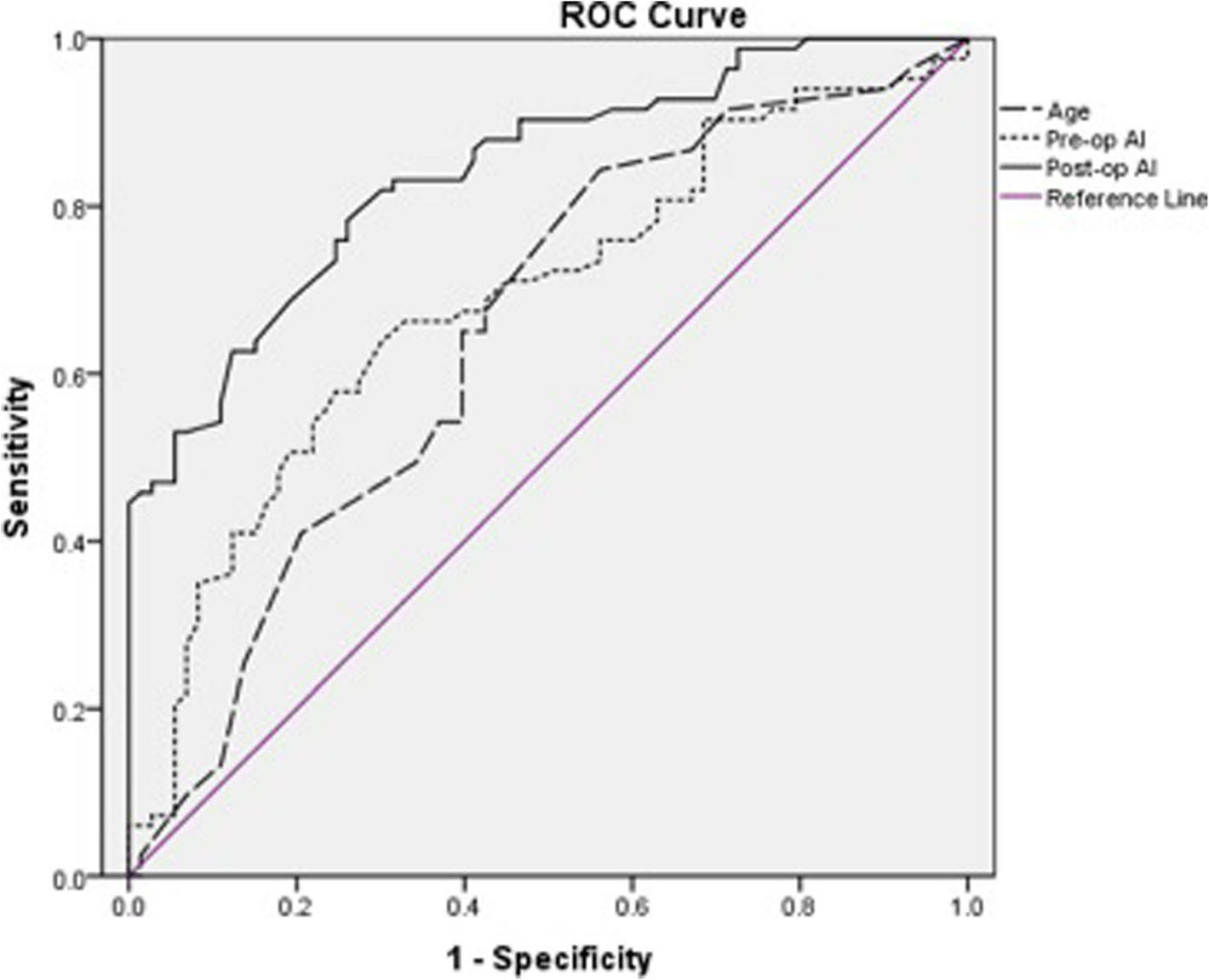
ROC was used to assess the sensitivity, specificity and diagnostic accuracy of identified risk factor, for predicting the CR failure. The pre-op AI has better sensitivity (0.783), specificity (0.740) and diagnostic accuracy (84.1%) than pre-op AI (0.639; 0.700; 68.8%) and age (0.651;185 0.603; 65.0%) for predicting the CR failure

ROC was used to assess the sensitivity, specificity and diagnostic accuracy of above identified risk factor, i.e., age, pre-op AI and post-op AI, for predicting the CR failure. The analysis of ROC curves indicates that post-AI has better reliability and goodness of fit than pre-op AI and age, because the sensitivity (0.783), specificity (0.740) and diagnostic accuracy (84.1%) were significantly better than pre-op AI (0.639; 0.700; 68.8%) and age (0.651;185 0.603; 65.0%) (*P* < 0.05) (Fig. 3).

## Discussion

The principle of the treatment for DDH is to establish a stable, concentric reduction of the hip to enable the subsequent hip development as early as possible, given the well-established correlation between residual dysplasia and the age of reduction. CR plays an essential role during the process of DDH treatment, especially the young children, with high success rate and low complications. Tschauner et al. [12] reported a safer, shorter and simpler way for the early DDH diagnosis and treatment using a sonographic hip screening programmer according to the Graf-method. In recent years, more attention has been drawn to the proper intervention strategy for DDH patients who are approaching or older than 18 months old. Normally, treatment can be CR followed by plaster casting, or performing OR as soon as possible once the diagnosis was established, since several studies indicated that older age might indicate poor outcome [5–7]. However, the timing for CR procedure is still a controversial issue among pediatric orthopedists. In our study, 107 pediatric patients (156 hips) with DDH in a single center were evaluated for the effect of CR with respect to different age groups, therefore to identify the risk factors of complications of CR and to discuss the possible indicators for failure of CR, especially in older age patients.

Compared with Group II and III, Group I showed significant difference about IHDI grade, but not Tönnis grade. Moreover, the ossific nucleus was not present in 34% hips. Comparing IHDI classification to Tönnis classification, Both Miao and Brandon et al. [13, 14] concluded that IHDI classification is more flexibly and better reflect the severity of the condition, especially for those cases without ossific nucleus of the femoral head.

For postoperative clinical attributes, the difference between Group I and Group II、III were statistically significant among post-op AI, which revealed that the older the child, the lower chance for the normalization of AI. The decrease of AI is a sign of gradual normalization of acetabular morphological structures under the condition of concentric reduction of the affected hip. Shin et al. [15] considered that an AI > 32° and CEA < 14° at the age of 3 years could serve as a guideline for osteotomy. Consistently, our results showed that if the post-op AI > 26.4°, CR was more likely to fail (84.1%). Pre-op AI also manifested with an obvious tendency to be fail if the value larger than 38.7° (68.8%). The ROC curve also showed that the age predictor for CR failure was the initially treatment age > 12.5 months (65%).

Treatment of DDH hinges heavily on the timing of diagnosis and treatment. The earlier the concentric reduction is achieved, the better outcomes it will be, due to the fact that the pathomorphology cannot proceed to extremely severe stages of luxation. The accompanying pathoanatomic obstacles greatly reduces the chance of a successful CR. Hence, early ultrasound screening using the Graf method in some countries is recommended to timely detect hip immaturity and pathologies and to provide the optimal approach [16]. Several studies reported older age at the time of CR showing a higher rate of complications or further corrective surgeries [5–7], while others not [8, 9, 17]. RAD in group III (older age) was found to be significantly higher, compared with Group I and II. Moreover, the result of univariable logistic regression models identified that age ≥ 18 months was the single significant risk factor for the occurrence of RAD (OR: 4.000; *p* = 0.012), which indicates the higher chance of RAD with the age of hip reduction increases. Other researches have indicated that in the case of lateral hip subluxation, the pressure on the femoral head becomes concentrated along the medial aspect of the head as the hip hinges along the edge of the acetabulum. The acetabular growth cartilage fills the acetabular floor and arrests its lateral growth, forming a progressively shallower and more oblique acetabulum [18, 19]. Therefore, we conclude that, for the dislocation patients, the risk of RAD must be brought for attention for children older than 18 months, which might in turn require FS to correct DDH.

Although in our study age does not play as a significant risk factor for AVN and re-dislocation. Similar to our results, it was also reported in previous studies [9, 17, 20] that age was not the risk factor of AVN after CR, while other studies gave the opposite conclusion [11, 21, 22]. The rate of AVN (15.4%) in this study falls into the range of the previously reported studies (10–33%) [7, 23–26]. The most common cause of AVN is the immobilization in a position that places excessive pressure on the femoral head. Thus, Ramsey et al. [27] recommended creating a “safe zone” to prevent AVN. In certain situation, an adductor tenotomy will increase the safe zone by allowing for a wider range of abduction, especially for patients with high Tönnis grade. Madhu et al. [28] collected data from nine studies and found out the most critical element of AVN was extreme abduction angle, whereas the ossification of the femoral head was not associated with AVN, which is consistent with our result and other studies [7, 29]. AVN is not associated with age nor other factors (sex, side, ossific nucleus etc.) in our cohort, but the IHDI IV was found to be a risk factor for both AVN and re-dislocation in univariable logistic regression analysis (OR: 2.524, *p* = 0.033; OR: 4.211, *p* = 0.004 l, respectively). For severe patients, CR is difficult to perform when extreme abduction is needed to stable reduction, which AVN might occur. The incidence of re-dislocation after CR is 14.7% in this study, which is similar to Sankar’s study (9%) [7]. Except from IHDI IV, the walking experience is also a risk factor for re-dislocation (OR: 2.524, *p =* 0.033). As the time proceeds, especially after the patient is capable of independent walk, a series of pathological changes of the affected hip will make CR more difficult, which, certainly, lowers the efficiency of CR [30, 31]. This is consistent with results in our study, namely, walking ability should be an important factor to evaluate at the time of treatment.

We also want to mention that a number of limitations exist in this study. First, a longer follow-up until early adulthood is more comprehensive, which may change the AVN and FS rate within the cohort, therefore affect the risk factors identified. Second, all the included cases had successful CRs at the initial attempt, which might bring a selection bias to the study. Third, the study was retrospective. More randomized controlled trials or large-scale case-control studies are required for further validation.

## Conclusion

In summary, we discovered that CR treatment initiated for patients older than 18 months of age produces higher rate of RAD and FS. The risk for re-dislocation is significantly associated with pre-op IHDI IV and walking. Pre-op IHDI IV was also found to be the risk factor of AVN. The threshold for age, pre-op AI and post-op AI values associated with an increased risk of failure are older than 12.5 months, lager than 38.7° and 26.4°, respectively. To avoid the CR failure, it is recommended that the parents of such children should be informed timely about the higher risk of treatment failure and further surgeries.

## Abbreviations

DDH: Developmental dysplasia of the hip
CR: Closed reduction
OR: Open reduction
AVN: Avascular necrosis
IHDI: International Hip Dysplasia Institute
AI: Acetabular index
RAD: Residual acetabular dysplasia
FS: Further surgeries
AUC: Area under the curve
ROC: Receiver operating characteristic curve
